# BRE Promotes Esophageal Squamous Cell Carcinoma Growth by Activating AKT Signaling

**DOI:** 10.3389/fonc.2020.01407

**Published:** 2020-08-11

**Authors:** Fujun Jin, Yexuan Zhu, Jingyi Chen, Rongze Wang, Yiliang Wang, Yanting Wu, Pengjun Zhou, Xiaowei Song, Zhe Ren, Jun Dong

**Affiliations:** ^1^Guangzhou Jinan Biomedicine Research and Development Center, College of Life Science and Technology, Institute of Biomedicine, Jinan University, Guangzhou, China; ^2^Integrated Chinese and Western Medicine Postdoctoral Research Station, Jinan University, Guangzhou, China; ^3^Department of Pathophysiology, School of Medicine, GHM Institute of CNS Regeneration, Jinan University, Guangzhou, China

**Keywords:** esophageal squamous cell carcinoma, brain and reproductive organ-expressed protein, AKT, apoptosis, cell cycle

## Abstract

Brain and reproductive organ-expressed protein (BRE) is aberrantly expressed in multiple cancers; however, its expression pattern in human esophageal squamous cell carcinoma (ESCC) and its role in ESCC progression remain unclear. In this study, we aimed to investigate the expression pattern of BRE in human ESCC and its role in ESCC progression. BRE was overexpressed in ESCC tissues compared with that in the adjacent non-tumor tissues. Forced expression of BRE was sufficient to enhance ESCC cell growth by promoting cell cycle progression and anti-apoptosis. Silencing of BRE suppressed these malignant phenotypes of ESCC cells. Mechanistic evaluation revealed that BRE overexpression activated the phosphorylation of AKT, and inhibition of the AKT pathway by MK2206 decreased the BRE-induced cell growth and apoptotic resistance in ESCC cells, highlighting the critical role of AKT signaling in mediating the effects of BRE. Moreover, the effects of BRE on ESCC cell growth and AKT activation were verified in a xenograft model *in vivo*. The present results show that BRE is overexpressed in ESCC tissues and contributes to the growth of ESCC cells by activating AKT signaling both *in vitro* and *in vivo* and provide insight into the role of BRE in AKT signaling and ESCC pathogenesis.

## Introduction

Esophageal squamous cell carcinoma (ESCC) is one of the most lethal malignant tumors and a serious threat to human health; it is the eighth most common malignant cancer and sixth leading cause of cancer-related mortality worldwide (1–3). Although diagnosis and treatment strategies have improved, the 5-year survival rate of patients with ESCC remains <20% (4). Late diagnosis, early metastasis, and a lack of targeted chemotherapeutic drugs contribute to the low survival rates of patients with ESCC (5). Therefore, it is important to identify regulatory factors contributing to ESCC progression to develop new drug intervention strategies for this lethal disease.

BRE is a stress-responsive gene that is expressed as multiple mRNA isoforms in human cells (6, 7). Differential expression of the BRE gene has been reported in response to various stress and biological signals, such as ionizing radiation and chorionic gonadotropin treatments (8). BRE is involved in numerous biological phenomena including apoptosis (9), DNA damage repair (10), cell differentiation (11), and tissue repair (12). BRE exerts anti-apoptotic effects by interacting with TNF-R1 and Fas to inhibit apoptotic activation (13, 14). Furthermore, BRE is indispensable for the integrity of the DNA damage repair-related BRCA1-A complex, wherein BRE interacts with MERIT40 to help form BRCA1 foci at DNA damage sites (15, 16). In humans, BRE overexpression is associated with the aggressiveness of liver and lung carcinomas (17, 18). However, BRE overexpression also helps predict favorable relapse-free survival in patients with breast cancer and adults with acute myeloid leukemia (19, 20). Thus, the role of BRE in tumorigenesis potentially depends on the tumor subtype. Chen et al. reported that BRE was overexpressed in a malignantly transformed esophageal carcinoma cell line in comparison with that in an immortalized human esophageal epithelial cell line (21). However, BRE expression in patients with ESCC, and its regulatory functions and role in ESCC progression remain unclear.

In this study, we aimed to investigate the expression pattern of BRE is human ESCC and its role in ESCC progression. Functional analyses of BRE knockdown or overexpression in four ESCC cells and mechanistic assays were carried out, followed by *in vivo* analysis using a mouse xenograft model to analyze the expression pattern of BRE in ESCC and determine its role in ESCC progression.

## Materials and Methods

### Cell Culture and Lentiviral Infection

ESCC cell lines KYSE140, KYSE450, KYSE510, Eca109, and TE-1 were obtained from the Chinese Academy of Sciences Cell Bank and maintained in RPIM-1640 medium (Gibco, USA) supplemented with 10% fetal bovine serum (Gibco) at 37°C and 5% CO_2_. For lentiviral infection, ESCC cells were infected with lentivirus at a multiplicity of infection of 20 in the presence of 5 μg/mL polybrene (Sigma, USA). Specific lentiviral short hairpin (shRNA) targeting the human BRE gene and scrambled control shRNA were purchased from Cyagen Biosciences (Guangzhou, China). The sequences were shBRE sense: TGT ACT TGT CAC CTC GAA T; shBRE antisense: ATT CGA GGT GAC AAG TAC A; Scramble sense: TTC TCC GAA CGT GTC ACG T; Scramble antisense: ACG TGA CAC GTT CGG AGA A. The stable BRE knockdown or overexpression ESCC cell lines were selected with 5 μg/ml of puromycin for 2 weeks and the stable cell lines were used in the subsequent cellular experiments.

### CCK-8 and Edu Assay

To analyze cell viability, ESCC cells with BRE knockdown or overexpression were plated into 96-well plates at a density of 1 × 10^4^ cells/well. After 24, 48, 72, or 96 h, the medium was replaced with medium containing 10% CCK-8 reagent and incubated at 37°C for 1 h, after which the absorbance was measured using a microplate reader at 450 nm. The OD_450_ value for each time point was used to generate a growth curve. The proliferation of ESCC cells was measured using a commercial Cell-Light Edu *in vitro* Kit (Ribobio, China) in accordance with the manufacturer's instructions.

### Apoptosis and Cell Cycle Assay

Apoptotic cells were analyzed using the Annexin V/PI Apoptosis Detection Kit (Keygentec, China). For cell cycle analysis, the cells were synchronized in G0/G1-phase by serum-starvation for 24 h and then released and harvested after 12 h. The cells were then collected and stained using the Cell Cycle Analysis Kit (Beyotime, China) in accordance with the manufacturer's instructions. A total of 2 × 10^5^ cells was counted via flow cytometry (Calibur, BD Biosciences, USA) and the data were analyzed using FlowJo software.

### Clone Formation Assay

To evaluate the clone formation potential of ESCC cells, 200 cells were plated into each well of a 6-well plate, and the plate was incubated at 37°C and 5% CO_2_ in an incubator for 2 weeks. The medium was replaced every 3 d. After 2 weeks, the clones were stained with 0.5% crystal violet for 30 min and enumerated.

### Western Blotting

Cultured ESCC cells and patient samples were homogenized in RIPA lysis buffer (Beyotime) in the presence of 1 mM PMSF (Beyotime) and lysed on ice for 30 min, followed by centrifugation at 14,000 × g for 10 min at 4°C to harvest the supernatant. Protein concentrations were determined using the BCA Protein Assay Kit (Beyotime) and normalized. Proteins were separated via SDS-PAGE and analyzed using the standard western blotting protocol. GAPDH was used as the internal control. The following primary antibodies were used: anti-BRE (#ab177960, 1:1000, Abcam, UK), anti-AKT (#2920, 1:1000, Cell Signaling Technology, USA), anti-p-AKT (#4060, 1:1000, Cell Signaling Technology), anti-mTOR (#2983, 1:1000, Cell Signaling Technology), anti-p-mTOR (#5536, 1:1000, Cell Signaling Technology), anti-PTEN (#9188, 1:1000, Cell Signaling Technology, USA), anti-p110 (#4249, 1:1000, Cell Signaling Technology), anti-p85 (#4292, 1:1000, Cell Signaling Technology), and anti-GAPDH (#5174, 1:1000, Cell Signaling Technology) antibodies.

### Quantitative Real-Time PCR Analysis

The total RNA was extracted from cultured ESCC cells and patient samples with TRIzol reagent (Invitrogen, USA) in accordance with the manufacturer's protocols. An equal amount total RNA was reverse-transcribed into cDNA using the PrimeScript RT Master Mix Kit (TAKARA, Japan). The q-PCR assays were performed using the CFX96 Touch Real-Time PCR Detection System (Bio-Rad, USA) using the SYBR Premix Ex Taq II Kit in accordance with the manufacturer's instructions (TAKARA). Relative gene expression levels were normalized to those of the housekeeping gene GAPDH. Gene-specific primer pairs used herein were the following: BRE sense, GAA GCT GCC CGT AGA TTT CA; BRE antisense, GTG GCT TCA GTG TCC TCA AA; PTEN sense, TAG ACC AGT GGC ACT GTT GT; PTEN antisense, TGG CAG ACC ACA AAC TGA GGA T; GADPH sense, AGC CTC AAG ATC ATC AGC AAT G; GADPH antisense, CAC GAT ACC AAA GTT GTC ATG GAT.

### Immunohistochemical Analysis

Patient ESCC tumor tissue and matched peri-tumor normal esophageal tissues were obtained from Sun Yet-Sen University Cancer Center. Written informed consent was obtained from all ESCC patients before the study. The use of the clinical specimens for research purposes was approved by the Ethics Committee of Jinan University. Tissue samples were fixed with 4% paraformaldehyde and embedded in paraffin and sectioned for immunohistochemical staining using the standard protocols. Primary antibodies against BRE were obtained from Abcam (ab177960, 1:50), and the staining results were scored by three pathologists without any information regarding tissue features. A semi-quantitative immunoreactive score (IRS) was used to evaluate the expression of the BRE proteins. Briefly, IRS was calculated by multiplying the staining intensity (graded as: 0 = no staining, 1 = weak staining, 2 = moderate staining, and 3 = strong staining) with the percentage of positively stained cells (0 = no stained cell, 1 = <10% of stained cells, 2 = 10–50% of stained cells, 3 = 51–80% of stained cells, and 4 = >80% of stained cells). An IRS <4 was defined as low expression, IRS between 4 and 8 was defined as medium expression, and IRS≤ 8 was defined as high expression (22, 23).

### Xenograft Models

To analyze the *in vivo* growth of ESCC cells, 2 × 10^5^ Eca109 or YSE140 cells were resuspended in 200 μl of 50% Matrigel PBS (Corning, USA) and injected subcutaneously into 5-week-old female BALB/c nude mice, six mice for each group. The tumor volume was measured every 3 days as described previously (24). After 3 weeks, the mice were euthanized, and the tumors were harvested. Mouse handling and experimental protocols were approved by the Experiment Animal Care Committee and the Ethics Committee of Jinan University (Guangzhou, China).

### Statistical Analysis

Data are expressed as the mean ± SEM values, and between-group comparisons were carried out using the paired samples Student's *t*-test for patients IHC samples scores or independent samples Student's *t*-test. The threshold for statistical significance was set to ^*^p < 0.05; ^**^p < 0.01.

## Results

### BRE Is Frequently Overexpressed in ESCC Tissue

**Figure 1:**
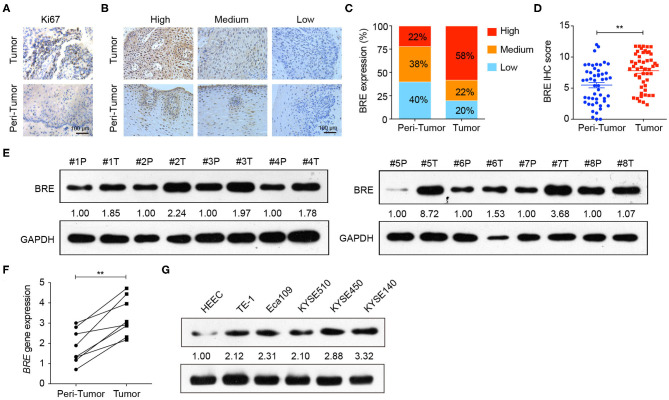
BRE was overexpressed in ESCC tissues. **(A)** Representative images of IHC analysis of Ki67 staining in ESCC and tumor-adjacent normal tissue. **(B)** Representative photographs of IHC analysis with high, medium, and low intensities of BRE staining in ESCC and tumor-adjacent normal tissue. **(C)** Immunohistochemistry score of BRE in 50 pairs of ESCC and peri-tumor tissues. **(D)** Percentage of samples with high, medium, and low BRE protein levels in 50 pairs of ESCC and peri-tumor tissues. **(E)** BRE protein levels in eight ESCC (T) and peri-tumor tissues (P); **(F)** *BRE* gene expression levels in eight paired ESCC and tumor-adjacent tissues. **(G)** BRE protein levels in five ESCC cell lines and normal esophageal epithelial cell lines HEEC. Data are expressed as mean ± SEM values; paired Student's *t*-test was used to evaluate significant differences, **p < 0.01.

To examine BRE expression in ESCC, immunohistochemical analysis was performed in 50 pairs of ESCC and matched tumor-adjacent normal tissue. The IHC analysis of the cell proliferation marker Ki67 showed that the proliferation of the tumor tissues was significantly higher than that of tumor-adjacent normal tissues (Figure 1A), thus identified the profile of the samples we collected. Based on the IHC density, the tissues samples were divided into three groups in accordance with BRE expression levels (high, medium, and low) (Figure 1B). The immunohistochemical analysis revealed that BRE was significantly upregulated in ESCC tissues than in the tumor-adjacent normal esophageal tissues (Figure 1C). Consistently, 58% of ESCC samples (29/50) displayed BRE upregulation, whereas only 22% (11/50) of peri-tumor normal esophageal samples displayed BRE upregulation (Figure 1D). Furthermore, we detected BRE expression in eight ESCC samples via western blotting and q-PCR analysis, as shown in Figures 1E,F, and BRE protein was clearly overexpressed in ESCC samples compared with that in the tumor-adjacent normal esophageal tissues. Finally, BRE expression in five ESCC cell lines and the normal human esophageal epithelial cell (HEEC) line was analyzed by western blotting; concurrent with clinical samples, BRE was also significantly upregulated in ESCC cell lines (Figure 1G).

### BRE Promotes ESCC Cell Proliferation

**Figure 2:**
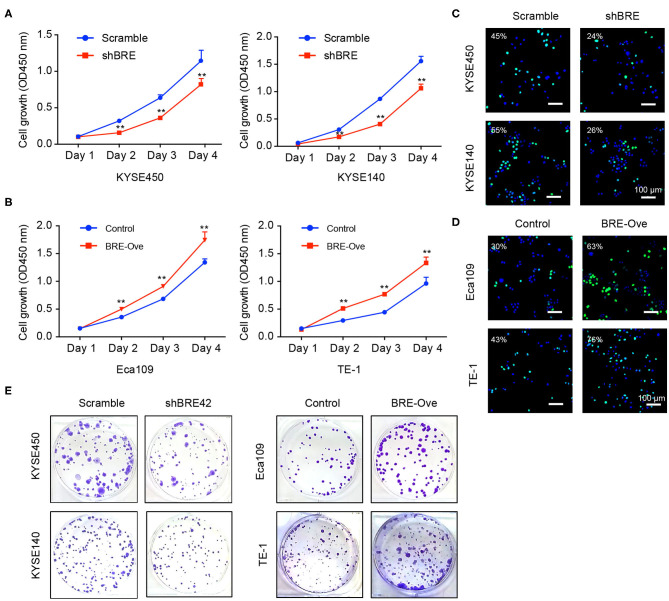
BRE promotes ESCC cell proliferation. **(A,B)** The viability of ESCC cell lines after BRE knockdown or overexpression was determined using the CCK-8 assay. Data are expressed as mean ± SEM; two-way ANOVA was used to evaluate significant differences, **p < 0.01; **(C,D)** Representative photographs of EdU-incorporated cells after BRE knockdown or overexpression. **(E)** Representative photographs of clone formation assays in different ESCC cell lines after BRE knockdown or overexpression.

**Figure 3:**
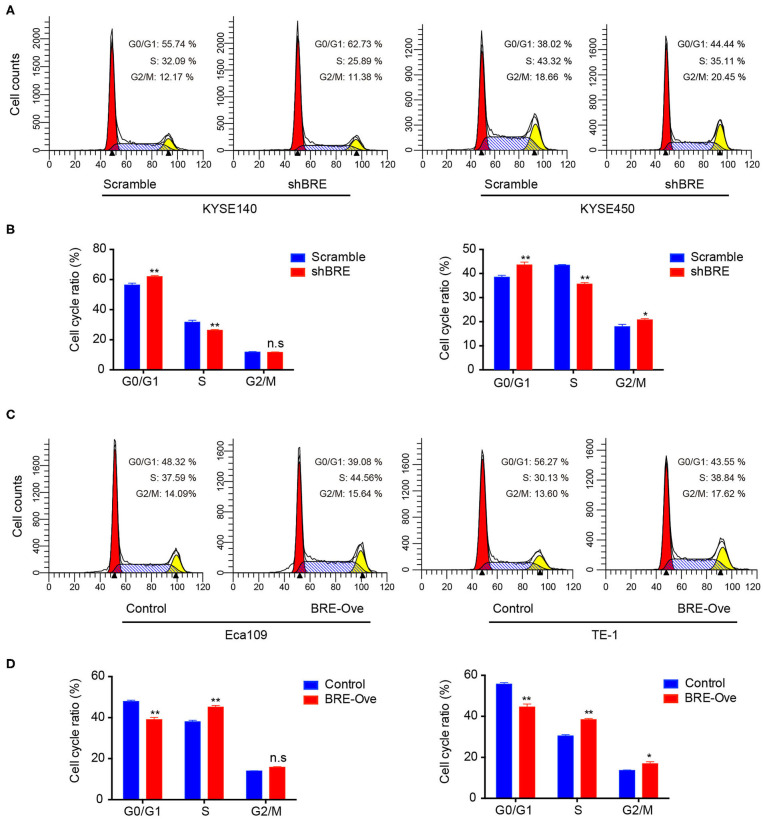
BRE promotes cell cycle progression of ESCC cells. Cell cycle distribution of ESCC cell lines after BRE knockdown **(A)** or overexpression **(C)** were analyzed via flow cytometry. **(B,D)** Quantitative data of the flow cytometry, *n* = 3; data are expressed as mean ± SEM. The two-way ANOVA was used to evaluate significant differences, *p < 0.05, **p < 0.01.

To investigate the functions of BRE in ESCC, we knocked down and overexpressed the BRE genes in ESCC cell lines, using lentiviruses. The CCK-8 assay revealed that the viability of two ESCC cell lines, KYSE450 and KYESE140, was significantly decreased when BRE was knocked down (Figure 2A), whereas BRE overexpression significantly increased the viability of the Eca109 and TE-1 ESCC cell lines (Figure 2B). Furthermore, the cell proliferation rate was analyzed using the EdU assay. As shown in Figure 2C, the knockdown of BRE expression considerably decreased the percentage of EdU-positive proliferating cells in KYSE450 and KYESE140 cell lines. However, BRE overexpression significantly promoted the proliferation of Eca109 and TE-1 ESCC cells (Figure 2D). Concurrently, the colony formation assay results showed that BRE knockdown inhibited self-renewal in ESCC cells, whereas BRE overexpression exhibited the opposite effects (Figure 2E). Meanwhile, the cell cycle analysis revealed that the knockdown of BRE in KYSE450 and KYESE140 cells significantly decreased the percentage of cells in the S phase and increased the percentage of cells in the G0/G1 phase (Figures 3A,B). In contrast, BRE overexpression significantly increased the percentage of Eca109 and TE-1 cells in the S phase (Figures 3C,D). These results show that BRE promotes cell cycle progression to induce the growth of ESCC cells.

### BRE Inhibits Apoptosis in ESCC Cells

**Figure 4:**
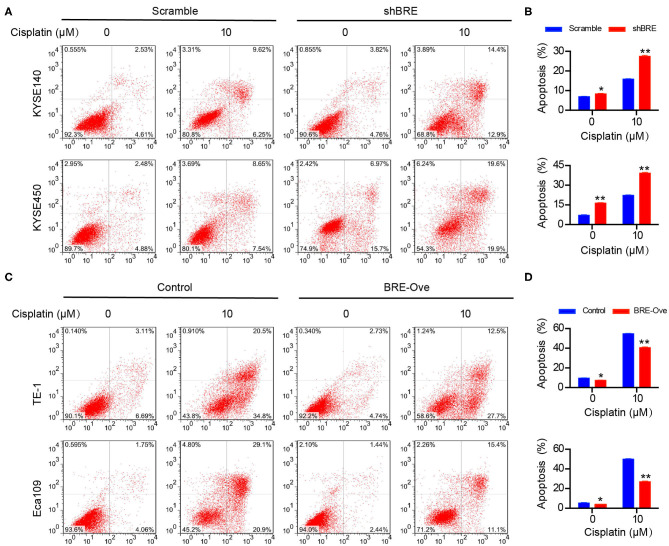
BRE influences apoptosis of ESCC cells. The apoptotic cells of different ESCC cell lines after **(A)** BRE knockdown or **(C)** overexpression were analyzed by flow cytometry. **(B,D)** Quantitative data of flow cytometry, *n* = 3; data are expressed as mean ± SEM. The two-way ANOVA was used to evaluate significant differences, *p < 0.05, **p < 0.01.

We performed the flow cytometry analysis to determine the effect of BRE on apoptosis in different ESCC cells. As shown in Figures 4A,B, BRE knockdown in KYESE140 and KYSE450 cells significantly increased the percentage of apoptotic cells from 6.9 to 8.5% in KYSE140 cells and from 7.2 to 16.5% in KYSE450 cells. Upon lentiviral overexpression of BRE, apoptotic cell death was considerably inhibited. BRE overexpression downregulated the percentage of apoptotic cells from 9.8 to 7.6% in TE-1 cells and from 5.6 to 3.9% in Eca109 cells (Figures 4C,D). Moreover, in the presence of apoptosis-inducing anti-tumor drug cisplatin, BRE knockdown more strongly induced apoptotic cell death in both cell lines. The percentage of apoptotic cells increased from 16.1 to 27.2% in KYSE140 cells and from 22.4 to 39.3% in KYSE450 cells (Figures 4A,B). As expected, BRE overexpression inhibited the apoptosis of ESCC cells, and the percentage of apoptotic cells decreased from 55 to 40% in TE-1 cells and from 50.1 to 27.1% in Eca109 cells (Figures 4C,D).

### BRE Regulates AKT Signaling to Modulate ESCC Growth and Apoptosis

**Figure 5:**
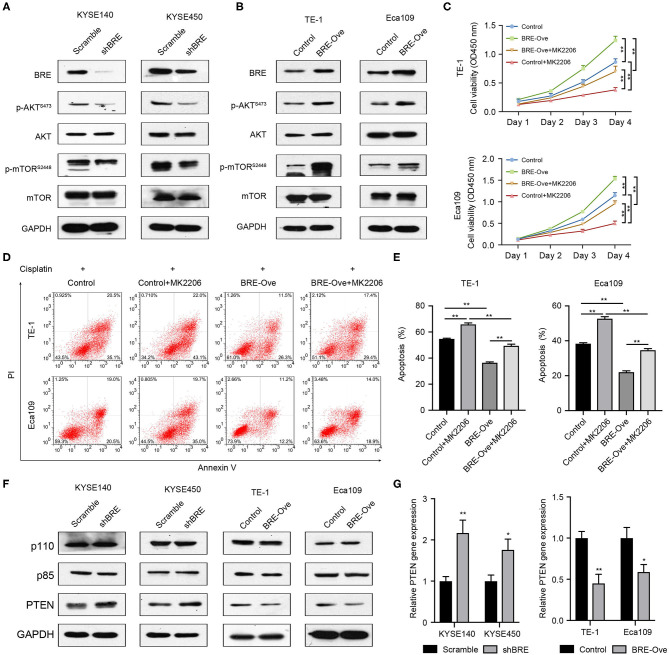
BRE positively modulates the AKT pathway. Expression of molecules of the AKT signaling pathway upon BRE knockdown **(A)** or overexpression **(B)** was analyzed by western blotting. **(C)** The viability of BRE overexpressed Eca109 and TE-1 cells with or without 10 μM MK2206, an AKT inhibitor, treatment were analyzed using the CCK-8 assay, *n* = 3. Data are expressed as mean ± SEM. The two-way ANOVA was used to evaluate significant differences, n.s, not significant, **p < 0.01. **(D)** Eca109 and TE-1 cells with or without BRE overexpression were treated with 10 μM cisplatin for 24 h; one BRE overexpression group was simultaneously treated with 10 μM MK2206, and the percentage of apoptotic cells was analyzed by flow cytometry. **(E)** Quantitative data of flow cytometry, *n* = 3. **(F)** Expression of molecules of the AKT signaling upstream regulators upon BRE knockdown or overexpression was analyzed by western blotting. **(G)** qPCR analysis of PTEN gene expression in BRE knockdown or overexpression ESCC cells, *n* = 3. Data are expressed as mean ± SEM; Student's *t*-test was used to evaluate significant differences, *p < 0.05, **p < 0.01.

AKT signaling is widely involved in regulating cancer cell growth and inhibition of apoptosis (25). Meanwhile, through analysis of the published microarray data we found that BRE knockdown downregulated the AKT signaling pathways (26). Therefore, we investigated the effect of BRE on the AKT signaling pathways through western blotting in four ESCC cell lines. As shown in Figure 5A, BRE knockdown inhibited the phosphorylation of AKT and its down-stream target mTOR without influencing the total protein level of both AKT and mTOR. However, when BRE was overexpressed in Eca109 and TE-1 cells, AKT and mTOR phosphorylation were considerably upregulated (Figure 5B). Furthermore, to determine whether AKT is responsible for BRE-induced cell growth and apoptotic resistance in ESCC cells, we used the widely used AKT inhibitor MK2206 to inhibit the activity of AKT in BRE-overexpressing cells. The results showed that AKT inhibition significantly diminished the cell growth (Figure 5C) and apoptotic resistance (Figures 5D,E) in both BRE-overexpressing Eca109 and TE-1 cells. Meanwhile, BRE overexpression also significantly reversed the cytotoxicity of the AKT inhibitors (Figures 5C–E). These results indicate that BRE could modulate the AKT pathway to regulate ESCC growth and survival. Next, to reveal the detailed mechanisms of action of BRE in AKT activation, we detected the expression levels of AKT upstream regulators. The WB experiment results showed that knockdown of BRE significantly increased the expression of PTEN, a negative regulator of AKT activation (Figure 5F). Contrarily, overexpression of BRE significantly decreased the expression of PTEN (Figure 5F). However, both knockdown and overexpression of BRE had no significant influence on the expression of PI3K, a positive regulator of AKT activation (Figure 5F). Furthermore, we found that BRE could regulate the expression of PTEN (Figure 5G). These results indicate that BRE could negatively regulate the expression of PTEN to modulate AKT activation.

### BRE Promotes ESCC Growth *in vivo*

**Figure 6:**
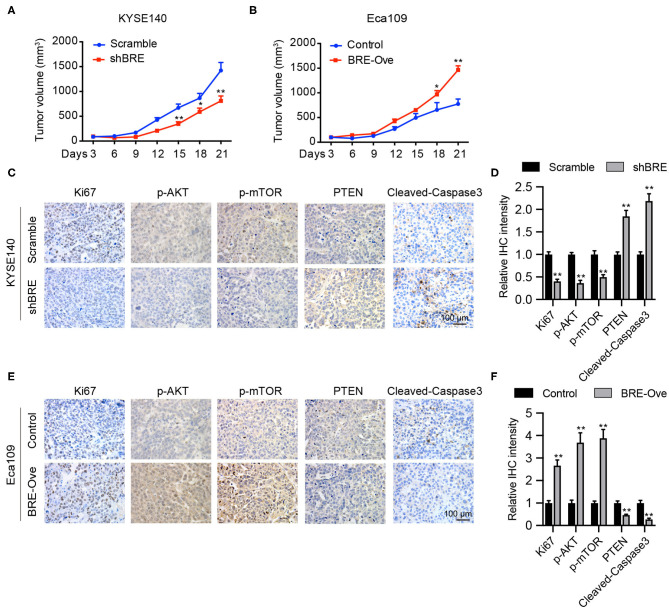
BRE promotes ESCC growth *in vivo*. Xenograft tumor growth curve of xenograft tumors generated via BRE knockdown in KYESE140 cells **(A)** or in BRE-overexpressing Eca109 cells **(B)**, *n* = 6, and data are expressed as mean ± SEM; two-way ANOVA was used to evaluate significant differences, *p < 0.05, **p < 0.01. Representative photographs of immunohistochemical analysis of Ki67, p-AKT, p-mTOR, PTEN, Cleaved-Caspase3 in xenograft tumor sections formed by BRE knockdown in KYESE140 cells **(C)** or BRE-overexpressing Eca109 cells **(E)**. **(D,F)** The immunoreactive areas in the IHC images were quantified using ImagePro Plus 6.0 software (Media Cybernetics, Silver Spring, MD). The integrated optical density (IOD) values were represented as the mean ± SEM. Student's *t*-test was used to evaluate significant differences, **p < 0.01.

We evaluated the function of BRE *in vivo*. KYSE140 cells with a stable BRE knockdown and Eca109 cells with stable BRE overexpression were subcutaneously injected into the flanks of nude mice to induce the development of xenograft tumors. Our data show that the *in vivo* growth of KYSE140 cells was greatly reduced upon BRE knockdown (Figure 6A). While, the growth of BRE-overexpressing Eca109 cells was accelerated in comparison with the control group (Figure 6B). Next, we evaluated the pathologic changes induced by BRE knockdown or overexpression through Ki67 IHC staining of the xenograft tumor sections (Figures 6C–F). The results showed that BRE considerably increased the percentage of Ki67+ cells and promoted ESCC growth *in vivo*. Finally, we detected the expression of p-AKT, p-mTOR, PTEN and cleaved-Caspase3 in xenograft tumors via IHC analysis. Our results showed that BRE knockdown considerably downregulated the expression of p-AKT and p-mTOR, but increased the expression of PTEN and apoptosis markers cleaved-Caspase3 in xenograft tumors formed by BRE knockdown KYSE140 cells (Figures 6C,D). In contrast, the expression of p-AKT and p-mTOR was significantly enhanced, whereas the expression of PTEN and cleaved-Caspase3 was notably decreased in BRE-overexpressed tissues (Figures 6E,F). Taken together, these results showed that BRE activated AKT signaling and inhibited the apoptosis of the ESCC xenograft tumor tissues and indicated that BRE plays an important role in promoting the growth of ESCC *in vivo*.

## Discussion

BRE is a highly conserved protein expressed nearly ubiquitously in all tissues in humans and mice (27) and is frequently upregulated in many tumors, thus regulating the development of different cancers (9, 28). Chen et al. reported that BRE was overexpressed in a malignantly transformed esophageal carcinoma cell line compared with that in an immortalized human esophageal epithelial cell line (21). However, the functions of BRE in human ESCC and its potential regulatory mechanism remain largely unclear.

In this study, we analyzed BRE protein expression in 50 paired ESCC and peri-tumor normal esophageal tissues *via* immunohistochemical analysis and reported that BRE is frequently upregulated in ESCC tissues compared with that in tumor-adjacent normal esophageal tissues. These results were verified by western blotting in eight paired ESCC and peri-tumor normal esophageal tissues. Furthermore, the q-PCR analysis revealed that BRE expression was consistently upregulated in ESCC tissues. Thus, BRE may play a similar role in ESCC and HCC (28) in regulating the growth and apoptosis of ESCC cells. Therefore, we performed knockdown and overexpression experiments to determine the role of BRE in ESCC progression *in vitro*. Our results showed that BRE knockdown significantly inhibited the growth of ESCC cells by inhibiting cell proliferation and cell cycle progression and inducing apoptosis. However, when BRE was ectopically overexpressed, ESCC cell growth was significantly increased. Furthermore, overexpression inhibited antitumor drug-induced apoptosis in ESCC cells, indicating that BRE promotes chemotherapeutic resistance in ESCC cells. Finally, we examined the functions of BRE in ESCC growth *in vivo* and found that BRE overexpression accelerates ESCC cell growth. These results show that BRE positively regulates ESCC cell growth both *in vitro* and *in vivo*. Furthermore, these results are consistent with those of studies on HCC and lung cancers (9, 17, 28).

Our results show that BRE positively modulates AKT phosphorylation in ESCC cells. BRE knockdown significantly downregulated p-AKT and p-mTOR, an important downstream target of p-AKT signaling. In contrast, both p-AKT and p-mTOR were significantly upregulated in BRE-overexpressing ESCC cells. Moreover, by treating BRE-overexpressed ESCC cells with an AKT inhibitor, we further confirmed that AKT signaling was required for cell growth and apoptotic resistance in BRE-overexpressing cells, which is consistent with studies reporting that AKT potently promotes ESCC cell progression and patient survival (29, 30). Tang et al. reported that the *Akt-3* gene was upregulated upon BRE knockdown in C2C12 cells (31). Our results show that BRE does not influence total AKT expression; however, it significantly promotes AKT phosphorylation in ESCC cells. These results show that AKT regulation by BRE depends on the cell type. Furthermore, the results indicated that BRE could inhibit the expression of the AKT upstream negative regulator PTEN, without any influence on the expression of the AKT upstream positive regulator PI3K. In our previous study, we had reported that BRE could negatively regulate the stability of p53 (32). Considering that PTEN was a target gene of the p53, we hypothesize that BRE may modulate the gene expression of PTEN through p53 in ESCC cells.

In conclusion, our study shows that BRE is frequently overexpressed in ESCC tissues, and overexpression of BRE in ESCC cells activated the AKT signaling pathway, thereby increasing ESCC cell growth and decreasing apoptosis. These data are potentially applicable for the development of ESCC interventions and treatments. However, the limitations of this study are the relatively small number of patients examined and lack of clinical data regarding the disease progression. Thus, the correlation between BRE expression and ESCC disease progression requires further analysis. Meanwhile, the detailed regulatory roles of BRE on AKT activation and PTEN expression also need further exploration.

## Data Availability Statement

The datasets generated for this study are available on request to the corresponding author.

## Ethics Statement

The studies involving human participants were reviewed and approved by The Ethics Committee of Jinan University. The patients/participants provided their written informed consent to participate in this study. The animal study was reviewed and approved by The Experiment Animal Care Committee and the Ethics Committee of Jinan University.

## Author Contributions

FJ, ZR, and JD: conceptualization and supervision. YZ, FJ, RW, and JC: investigation. FJ, YWa, and YWu: formal analysis. PZ and XS: resources. FJ: original draft preparation. ZR and JD: manuscript review and editing. All authors read and approved the final manuscript.

## Conflict of Interest

The authors declare that the research was conducted in the absence of any commercial or financial relationships that could be construed as a potential conflict of interest.
